# Use and effects of implementation strategies for practice guidelines in nursing: a systematic review

**DOI:** 10.1186/s13012-021-01165-5

**Published:** 2021-12-04

**Authors:** Christine E. Cassidy, Margaret B. Harrison, Christina Godfrey, Vera Nincic, Paul A. Khan, Patricia Oakley, Amanda Ross-White, Hilary Grantmyre, Ian D. Graham

**Affiliations:** 1 School of Nursing, Dalhousie University, 5860 University Ave., Halifax, NS B3H 4R2 Canada; 2 School of Nursing, Queen’s University, 92 Barrie Street, Kingston, ON K7L 3J9 Canada; 3 Li Ka Shing Knowledge Institute of St. Michael’s Hospital, 209 Victoria Street, Toronto, ON M5B 1W8 Canada; 4 National Research Council Canada, Institute for Information Technology, 46 Dineen Drive, Fredericton, NB E3B 9W4 Canada; 5 Queen’s University Library, Queen’s University, 18 Stuart Street, Kingston, ON K7L 3N6 Canada; 6 School of Epidemiology and Public HealthSchool of Nursing, University of Ottawa, Ottawa Hospital Research Institute, 501 Smyth Road, Ottawa, ON K1H 8L6 Canada

**Keywords:** Clinical practice guidelines, Nursing, Implementation, Implementation strategies, Systematic review

## Abstract

**Background:**

Practice guidelines can reduce variations in nursing practice and improve patient care. However, implementation of guidelines is complex and inconsistent in practice. It is unclear which strategies are effective at implementing guidelines in nursing. This review aimed to describe the use and effects of implementation strategies to facilitate the uptake of guidelines focused on nursing care.

**Methods:**

We conducted a systematic review of five electronic databases in addition to the Cochrane Effective Practice and Organization of Care (EPOC) Group specialized registry. Studies were included if implementation of a practice guideline in nursing and process or outcome of care provided by nurses were reported. Two reviewers independently screened studies, assessed study quality, extracted data, and coded data using the EPOC taxonomy of implementation strategies. For those strategies not included in the EPOC taxonomy, we inductively categorized these strategies and generated additional categories. We conducted a narrative synthesis to analyze results.

**Results:**

The search identified 46 papers reporting on 41 studies. Thirty-six studies used a combination of educational materials and educational meetings. Review findings show that multicomponent implementation strategies that include educational meetings, in combination with other educational strategies, report positive effects on professional practice outcomes, professional knowledge outcomes, patient health status outcomes, and resource use/expenditures. Twenty-three of the 41 studies employed implementation strategies not listed within the EPOC taxonomy, including adaptation of practice guidelines to local context (*n* = 9), external facilitation (*n* = 14), and changes to organizational policy (*n* = 3). These implementation strategies also corresponded with positive trends in patient, provider, and health system outcomes.

**Conclusions:**

Nursing guideline implementation may benefit from using the identified implementation strategies described in this review, including participatory approaches such as facilitation, adaptation of guidelines, and organizational policy changes. Further research is needed to understand how different implementation strategy components work in a nursing context and to what effect. As the field is still emerging, future reviews should also explore guideline implementation strategies in nursing in quasi or non-experimental research designs and qualitative research studies.

**Supplementary Information:**

The online version contains supplementary material available at 10.1186/s13012-021-01165-5.

Contributions to the literature

- Findings from this review may inform nursing implementation researchers and practitioners in selecting strategies that facilitate the uptake of practice guidelines in nursing.
- This review identified additional implementation strategies similar to reviews in other health disciplines, including facilitation, guideline adaptation to the local context, changes to organizational policies, and use of a participatory approach.
- Future implementation science research in nursing, using qualitative, quantitative, and mixed methods research designs, is needed to help move the field beyond educational strategies and understand what works, for whom, and in what context.

## Background

Implementing evidence into health care practice is essential for improving outcomes for patients, providers, and the health care system [1, 2]. However, recent research estimates that on average, only 60% of care is consistent with evidence or consensus-based guidelines, 30% of care is either ineffective or low value, and 10% of care is harmful [3].

Nurses are the largest group of practitioners in health care systems, and thus have considerable potential to translate evidence into practice and influence patient and health system outcomes. A significant amount of international research, theory/framework design, policy, and education has been developed to advance the application of evidence-based nursing practice [4]. Notably, practice guidelines have emerged as a key tool for translating evidence into practice [5].

Practice guidelines are developed from systematic reviews of current evidence and offer graded recommendations that reflect best practice [5]. Guidelines have shown to be an effective strategy for improving health outcomes and processes of care in medicine [2, 6]. Despite these benefits, implementation of guidelines is both complex and varied [7–9]. Development of practice guidelines does not necessarily guarantee health care provider adoption and adherence in practice. A scoping review of barriers to guideline implementation in health care settings identified barriers related to personal factors (i.e., knowledge and attitudes), guideline-related factors (i.e., evidence, complexity, accessibility, and applicability of the guidelines), and external-factors (i.e., organizational constraints, resources, social and clinical norms) [10]. Tailored implementation strategies are needed to address these barriers and support uptake of guidelines in practice to impact patient and health system outcomes [11].

There is a need to fully identify the range of implementation strategies as well as the most effective strategies to improve the utility of guidelines in nursing practice. Previous systematic reviews have evaluated the effectiveness of implementation strategies, primarily in the medical domain [2, 12] and among allied health professionals, including rehabilitation [13], physiotherapy [14], dentistry [15], and pharmacy [16]. However, as Thompson and colleagues [17] point out, the nature and social structure of nursing work differs greatly from medicine and allied health professions. Nurses typically work in teams and in settings with procedures and protocols thus, not necessarily making sole decisions about care. Often the focus of implementation in nursing needs to be with a group and/or organization in addition to the individual [18]. As such, drawing conclusions about the effectiveness of interventions aimed at physician/allied health practice to guide implementation in nursing practice may not be appropriate. The purpose of this systematic review is to describe the use and effects of implementation strategies to facilitate uptake of guidelines focused on nursing care.

A recent systematic review examined implementation strategies used to implement nursing guidelines in daily practice [19]. This review identified 54 articles that employed a variety of implementation strategies with a majority of studies (68%) reporting a positive effect on patient-related nursing outcomes or guideline adherence [19]. Our systematic review builds on these findings by replicating a similar approach and further examining the effect of implementation strategies on nurses’ knowledge and practice, patient health status outcomes, and resource use/expenditures. Replication of systematic reviews is often disregarded, done poorly, or done unnecessarily [20]. Lack of or poor replication can lead to development and implementation of policies, guidelines, or practices that are based on weak evidence. Karunananthan et al. [21] describes two types of systematic review replication, including (a) the repetition of the same population, intervention, comparison, or outcome (PICO) using the same or very similar methods to a previous review, or (b) broadening or narrowing the PICO of a previous review. Our systematic review employs the second type of replication whereby the PICO is overlapping with the Spoon et al. [19] review but with a broader outcome focus and narrower study design focus. Collectively, these reviews add to the knowledge base on effective guideline implementation strategies in nursing.

## Methods

This review protocol was originally registered with Cochrane Effective Practice and Organization of Care (EPOC). The search strategy and screening methods followed Cochrane systematic review and meta-analysis guidance (see Supplemental File 1 for our a priori EPOC review methods). This initial review generated a heterogeneous set of papers with a wide variety of implementation strategies (i.e., mode of delivery, dose, frequencies), and outcome measures. Although the data described specific strategies, it was not helpful for interpreting the overall effectiveness and utility of implementation strategies in nursing or the next steps for future research in nursing implementation. We were challenged to make sense of the data in a useful way for practice and to move the state of the science forward. Following this initial analysis, we identified several ad hoc discoveries as equally, if not more, important for nursing implementation science and future reviews. We therefore conducted a narrative review of the included papers with a different lens than originally intended to more fully describe the findings related to implementation strategies for nursing. The following methods and results reflect this narrative approach to the review. We completed the review in accordance with the Preferred Reporting for Systematic Reviews and Meta-Analyses (PRISMA) statement and checklist. See Supplemental File 1 for deviations from the initial protocol.

### Information sources and search strategy

Five electronic databases (Medline, EMBASE, CINAHL, PsycINFO, AMED) and the Cochrane EPOC registry were systematically searched using a search strategy developed with nursing library scientists at Queen’s University. The team used search terms and medical subject headings (MeSH) relevant to “clinical guidelines” AND “implementation” AND “nursing” AND “randomized controlled trial” (Supplemental File 2). The search was run up to September 30th, 2020 with no restrictions. We also used the search strategies developed by Grimshaw and colleagues [22] for their investigation of the effectiveness and efficiency of guideline dissemination and implementation strategies in the context of medicine. These search strategies were adjusted to focus on nursing and rerun against MEDLINE, CINAHL, and EMBASE. We also scanned reference lists of papers identified for inclusion for any additional references not captured.

Eligibility criteria.

Inclusion criteria for this review were that studies had to (a) be written in English and published in a peer-reviewed journal, (b) use a randomized controlled trial (RCT) design, and (c) evaluate the implementation of a guideline targeted to nurses or a multidisciplinary team with a focus on nursing outcomes. Implementation strategies are defined as “methods or techniques used to enhance the adoption, implementation, and sustainability of a clinical program or practice” [23]. Guidelines are defined as “systematically developed statements to assist practitioner and patient decisions about appropriate health care for specific clinical circumstances” [17]. A more recent definition includes benefits and harms (but no longer includes the goal of assisting practitioner and patient decisions); “Clinical practice guidelines are statements that include recommendations intended to optimize patient care that are informed by a systematic review of evidence and an assessment of the benefits and harms of alternative care options” [5]. Substitute terms for practice guidelines included “protocol,” “standard,” “algorithm,” and “clinical pathway.” The primary comparator was “usual practice” or “usual care” which indicates no distinct implementation strategy was used to change nursing practice or a traditional approach to dissemination was used (e.g., guidelines were available for use in practice setting, nurses received a copy of the guidelines). Categories of nurses included advanced practice nurses (APN); nurse practitioners (NPs); clinical educators (CE); clinical nurse specialists (CNS); registered nurses (RN); and licensed practical nurses (LPN)/registered practical nurses (RPN).

We excluded studies that were (a) not a RCT design; (b) abstracts, conference proceedings; (c) targeting implementation strategies to patients, administrators, and other health care providers (when outcomes could not be attributed to nurses); and (d) not focused on implementing practice guidelines.

### Outcomes

Outcomes of interest were primary outcomes focused on process or outcome of care provided by nursing professionals. Outcomes were grouped into five categories (professional knowledge, professional practice, patient health status outcomes, resource use, and expenditures). Professional knowledge outcomes related to level of nursing knowledge. Professional practice outcomes related to nursing process of care (i.e., adherence to practice guidelines). Patient health status outcomes included physical health and treatment outcomes (i.e., pain, quality of life, incontinence). Economic outcomes related to resource use and measured costs and cost savings (expenditures) associated with guideline implementation [24].

### Study selection

Two reviewers independently screened the titles and abstracts for inclusion in Covidence [25]. Next, two reviewers independently screened the full-text articles against the inclusion criteria. When there was disagreement, a third and independent reviewer assessed the study. Where separate papers reported on different aspects of the same study (for example, one paper describing the effect of the intervention on professional practice and another, the effect on patient health status outcomes), we treated them as one study with two companion papers.

### Data abstraction

Two reviewers independently abstracted data from included studies using a standardized form adapted from the EPOC data collection checklist. The following data were extracted from each eligible full-text study: (a) study design, (b) participants, (c) setting, (d) data collection methods, (e) practice guideline, (f) use of theory, (g) types of implementation strategies, (h) outcome measures, and (i) study results. Two reviewers independently piloted the data extraction form with two studies and revisions were made. Where there was disagreement in data abstraction, a third and independent reviewer assessed the study and resolved the conflict. For those studies where separate papers reported on different outcomes of the same study, only one data abstraction template was completed.

### Categorization of implementation strategies

We initially used the Cochrane Effective Practice and Organization Care Review Group (EPOC) taxonomy to describe the implementation strategies included in this review. The EPOC taxonomy has been used in previous reviews of implementation strategies with a similar scope but with different practice settings [16, 26]. To classify implementation strategies, we started by deductively categorizing strategies into the EPOC taxonomy’s section on implementation strategies [27]. Next, for those strategies not included in the EPOC taxonomy, we used an inductive thematic analysis approach to group these strategies and generate additional implementation strategy categories [28].

### Study quality

Two reviewers independently assessed the risk of bias using the EPOC Risk of Bias 2.0 checklist in Covidence [29]. For each of the included studies, risk of bias was assessed as a judgment of high, low, or unclear risk across nine domains. Discrepancies were resolved by consensus with a third reviewer when necessary.

### Data analysis

We conducted a narrative synthesis after identifying methodological and clinical heterogeneity in the studies of this review; this indicated that meta-analysis was not appropriate. A narrative synthesis allows for a description of implementation strategies and their effects in achieving outcomes for guideline implementation in nursing [30]. The frequency of each EPOC taxonomy category, outcome measure category, and outcome effect is reported. For outcome effect, we describe whether the outcomes were reported as a statistically significant positive effect or had no effect. Separate comparisons were made for categories of implementation strategies and compared to grouped study results to determine whether they were related to positive and significant improvement in: professional knowledge outcomes, professional practice outcomes, patient health status outcomes, resource use outcomes, and/or expenditure outcomes.

## Results

### Study selection

**Fig. 1:**
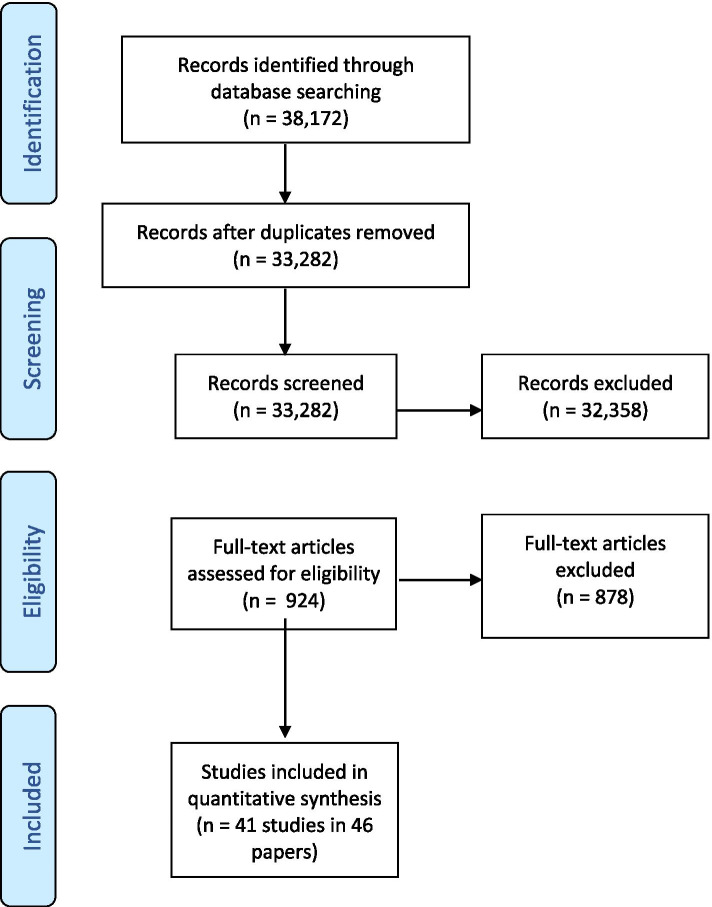
PRISMA flow diagram

All database searches and hand-searching of reference lists of included studies yielded a total of 38,172 citations. No studies were found using the Grimshaw et al. (22) search strategy. After removal of 4890 duplicates, 33,282 citations were screened, and 924 potential articles were identified. From this set, 878 articles did not meet our inclusion criteria and were excluded from analyses. The majority were excluded for not using an experimental study design (RCT), not implementing a guideline, and not including a nursing population. Forty-one studies (reported in 46 papers due to five companion reports) met final inclusion criteria (Fig. 1). All studies included were conducted between January 1996 and September 2020.

### Study characteristics

Full details of study-level characteristics are described in Table 1. The final set included 41 randomized controlled trials (RCTs), of which 24 were reported to be cluster randomized controlled trials (cRCT). The number of intervention arms ranged from two to three (two arms = 36 studies, three arms = 5 studies). In total, the 41 studies included a minimum of 5429 nurses (2 studies did not specify the number of participating nurses). In addition, a minimum of 231,218 patients were implicated in 27 studies, with 14 studies not reporting on the number of participating patients.

**Table 1:** Characteristics of included studies

Author	Study design	Participants: healthcare providers	Participants: patients	Setting	Practice guidelines
Ammerman, 2003 (USA)	RCT	Nurses	Food for Heart program patient	Hospital—Outpatient	Dietary counseling for hypercholesterolemia
Charrier, 2008 (Italy)	cRCT	Nurses	Adult inpatient	Hospital—Inpatient	Prevention of pressure lesions and the management of peripheral and central venous catheters
Cheater, 2006 (UK)	cRCT	Nurses	Adult outpatient	Hospital—Outpatient	Management of urinary incontinence
Daniels, 2005 (USA)	RCT	Nurses, Physicians	Adult outpatient	Hospital—Outpatient	Asthma care management
Day, 2001 (UK)	RCT	Nurses	Adult intensive care inpatient	Inpatient—Hospital	Endotracheal suctioning
Donati, 2020 (Italy)	cRCT	Nurses	Medical-surgical	Inpatient—Hospital	Standard precautions
Elliott, 1997 (USA)	cRCT	Nurses	Oncology patient	Community Primary Care Clinic	Cancer pain management
Evans, 1997(USA)	cRCT	Nurses, Physicians	Pediatric inpatient	Hospital—Outpatient	Asthma care management
Fairall, 2005, 2010 (South Africa)	cRCT	NP	Adult outpatient	Community Primary Care Clinic	Tuberculosis case detection and respiratory care
Feldman, 2004 (USA)	RCT	Nurses	Chronic heart failure patient	Hospital—Outpatient	Heart failure management
Friese 2019 (USA)	cRCT	Nurses	Oncology patients	Hospital—Inpatient	Hazardous Drug Handling
Haegdorens, 2018 & 2019 (Belgium)	cRCT	Nurses	Medical-surgical	Hospital—inpatient	Early warning score
Harrison, 2000 (South Africa)	RCT	Nurses	Community clinic patient	Community	Sexually transmitted infection management
Hödl, 2019 (Austria)	cRCT	Nurses	Nursing home resident	Nursing Home	Urinary incontinence management
Hodnett, 1996 (Canada)	cRCT	Nurses	Labor and delivery patients	Hospital—Inpatient	Intrapartum nursing practice
Jansson, 2014(Finland)	RCT	Nurses	Adult intensive care inpatient	Hospital—Inpatient	Prevention of ventilator-associated pneumonia
Jansson, 2016a, 2016b (Finland)	RCT	Nurses	Adult intensive care inpatient	Hospital—Inpatient	Prevention of ventilator-associated pneumonia
Kalinowski, 2015 (Germany)	RCT	Nurses	Nursing home resident	Nursing Home	Nonpharmacological pain management
Kaner, 2003 (UK)	cRCT	Nurses	Adult outpatient	Community Primary Care Clinic	Brief alcohol intervention
Köpke, 2012 (Germany)	cRCT	Nurses	Nursing home resident	Nursing Home	Use of physical restraint
Lozano, 2004 (USA)	RCT	Nurses, Physicians	Pediatric, asthmatic patient	Hospital—Outpatient	Pediatric chronic asthma care
Mayou, 2002 (UK)	RCT	Nurses	Adult heart failure inpatient	Hospital—Inpatient	Early rehabilitation after myocardial infarction
McDonald, 2005 (USA)	RCT	Nurses	Adult outpatient	Hospital—Outpatient	Pain management
Moon, 2015 (South Korea)	RCT	Nurses	Adult intensive care inpatient	Hospital—Inpatient	Delirium prevention
Murtaugh, 2005 (USA)	RCT	Nurses	Adult cardiology outpatient	Hospital—Outpatient	Heart failure disease management
Naylor, 2004 (USA)	RCT	Nurses	Adult cardiology inpatient	Hospital—Inpatient	Transitional care of older adults hospitalized with heart failure
Noome, 2017 (Netherlands)	RCT	Nurses	Adult inpatient	Hospital—Inpatient	Nursing end-of-life care
Pagaiya, 2005 (Thailand)	RCT	Nurses	Adult and pediatric outpatient	Community Primary Care Clinic	Children: Acute respiratory infection and diarrhea Adults: Diazepam prescribing and standard management of diabetes
Parker, 1995(USA)	RCT	Nurses, NP	Adult, long term care patient	Long-term care facility	Diabetes management
Premaratne, 1999 (UK)	RCT	Nurses	Community clinic patient	Health care clinic	Asthma management
Rood, 2005 (Netherlands)	RCT	Nurses	Adult, inpatient	Hospital—Inpatient	Glucose regulation
Ruijter, 2018 (Netherlands)	RCT	Nurses	Adult, outpatient	Community Primary Care Clinic	Smoking cessation
Snelgrove-Clarke, 2015 (Canada)	RCT	Nurses	Adult, low risk labor and delivery patient	Hospital—Inpatient	Fetal health surveillance
Titler, 2009; Brooks, 2008 (USA)	RCT	Nurses, Physicians	Older adults	Hospital—Inpatient	Acute pain management
Tjia, 2015 (USA)	cRCT	Nurses	Nursing home residents	Long term care	Antipsychotic prescribing
Vallerand, 2004 (USA)	RCT	Nurses	Adult outpatient	Hospital—Outpatient	Cancer pain management
Van Gaal, 2011a; 2011b (Netherlands)	RCT	Nurses	Older adults	Long term care and Hospitals—inpatient	Patient care guidelines to prevent adverse events including: pressure ulcers, urinary tract infections and falls
VonLengerke, 2017 (Germany)	RCT	Nurses, Physicians	Adult intensive care inpatient	Hospital—Inpatient	Hand hygiene
Weiss, 2019 (USA)	cRCT	Nurses	Adults, medical surgical	Hospital—Inpatient	Discharge Readiness Assessment
Wright, 1997 (USA)	RCT	Nurses	Adult inpatient	Hospital—Inpatient	Universal precautions-related behaviors
Zhu, 2018 (China)	RCT	Nurses	Adult, outpatient	Community Primary Care Clinic	Hypertension management

Eighteen studies were conducted in North America (16 in the USA, 2 in Canada), 5 in the UK, 13 in continental Europe (4 in the Netherlands, 3 in Germany, 2 in Finland, 2 in Italy, 1 in Austria, 1 in Belgium), 2 in South Africa, and 3 in Asia (1 in Thailand, 1 in China, and 1 in South Korea) (Table 1).

Key guideline topics included respiratory (asthma, pneumonia) (*n* = 7), heart conditions (*n* = 5), pain (*n* = 3), cancer (*n* = 2), diabetes (*n* = 2), pressure ulcer prevention (*n* = 2), maternity care (*n* = 2), and urinary incontinence (*n* = 2) (Table 1). Various nursing practices and health care settings were identified, including intensive care units, nursing homes, and in the community. The use of theory to inform the intervention and/or implementation strategy was used in 17 studies, including identifying behavioral and environmental determinants, identifying intervention targets, selecting implementation methods and delivery strategies, and informing measurement and evaluation.

### Risk of bias assessments

Methodological quality of the studies varied (Table 2). Most of the studies had a low risk of bias in their allocation concealment (*n* = 33, 80%), baseline outcome measurements (*n* = 36, 88%) and baseline characteristic similar (*n* = 32, 78%), as well as a low risk in other sources of bias (*n* = 37, 90%). Two-thirds of the studies completed high-quality (low risk) random sequence generations (*n* = 27, 66%), while a number of studies had an unclear risk of randomization techniques (*n* = 14, 34%). Knowledge of the allocated interventions was adequately prevented in half the studies (*n* = 21, 50%), while in the rest of the cases it was not clear (*n* = 19, 46%), or not prevented (*n* = 2, 5%). Compared to other categories, a higher level of risk was found for incomplete outcome data (*n* = 11, 27%) and in protection against contamination (*n* = 7, 17%); however, the majority were low risk of bias in these categories (*n* = 31, 76% and *n* = 29, 71%, respectively). Lastly, the majority of studies had unclear (*n* = 18, 44%) and low risk of bias (*n* = 24, 59%) for selective outcome reporting.

**Table 2:**
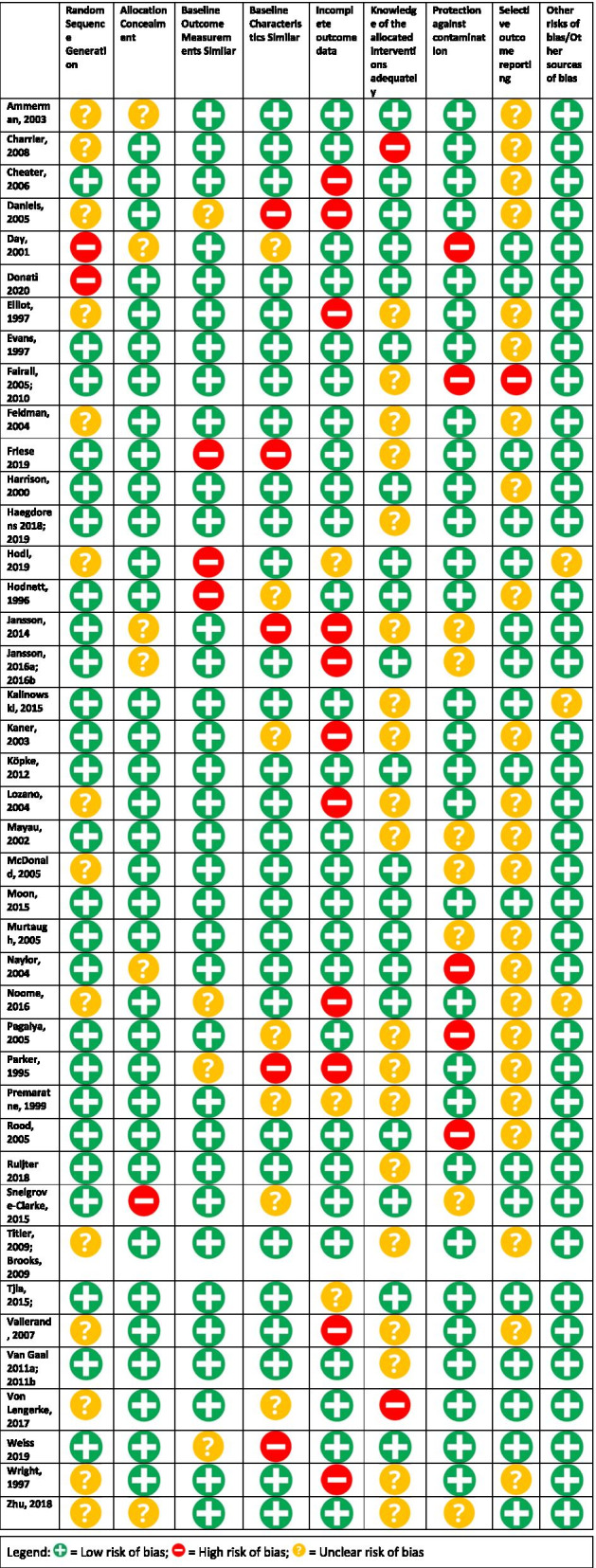
Risk of bias of included studies

### Implementation strategies used

Table 3 reports on implementation strategies used across all studies. Of the 41 studies, a total of 152 strategies were used. Multi-component implementation strategies were most commonly used (*n* = 36 studies) with only five studies reporting single component strategies.

**Table 3:** Summary of implementation strategies used in included studies and reported outcomes

Study author, date (country)	Implementation strategies from EPOC taxonomy									Implementation strategies not included in EPOC taxonomy				Outcomes
	Educational materials	Educational meetings	Local consensus process	Educational outreach visits	Local opinion leaders	Patient mediated interventions	Audit & feedback	Tailoring interventions	Reminders	Adaptation of CPG to local context	External facilitation	Changes to Org Policy	Multi-faceted participatory Approach	
Ammerman, 2003 (USA)		***X***												**Ø Patient health status** (attitudes/practices to prevent hypercholesterolemia; blood lipids, weight and dietary choices)
Charrier, 2008 (Italy)							***X***				***X***			**+ Professional practice** (adoption of guideline practices)
Cheater, 2006 (UK)	***X***	***X***		***X***			***X***	***X***						**Ø Professional practice** (adherence to urinary incontinence assessment guidelines)
Daniels, 2005 (USA)	***X***	***X***	***X***							***X***	***X***			**+ Professional practice** (compliance with asthma care guidelines)
Day, 2001 (UK)		***X***												**+ Professional knowledge** (knowledge of endotracheal suctioning) **+ Professional practice** (ability to practice guideline recommendations effectively)
Donati, 2020 (Italy)		***X***					***X***							**+ Professional practice** (hand hygiene)
Elliott, 1997 (USA)	***X***	***X***		***X***	***X***			***X***			***X***			**Ø Professional knowledge** (knowledge of cancer pain management, pain intensity)
Evans, 1997 (USA)	***X***	***X***	***X***	***X***							***X***			**+ Professional practice** (identification/treatment of asthma) **+ Patient health status** (continuity of care, patient education by professionals)
Fairall, 2005, 2010 (South Africa)	***X***		***X***	***X***				***X***		***X***				**+ Professional practice** (Increased case detection, improved therapy/practices by professionals, improved prescribing) **Ø Expenditures** (cost effectiveness)
Feldman, 2004(USA)	***X***	***X***		***X***						***X***	***X***			**+ Professional practice** (quality of life, satisfaction with home care services, survival at 9 days) **+ Resource use** (emergency department use, hospital admissions)
Friese, 2019 (USA)	***X***						***X***	***X***	***X***			***X***		**Ø Professional knowledge** (knowledge of PPE) **Ø Professional practice** (PPE use)
Haegdorens 2018, 2019 (Belgium)		***X***												**+ Professional practice** (frequency and quality of vital signs) **Ø Patient health status** (unexpected death, cardiac arrest, unplanned ICU admission
Harrison, 2000 (South Africa)	***X***	***X***		***X***						***X***				**+ Professional practice** (quality of case management)
Hödl, 2019 (Austria)	***X***	***X***												**+ Patient health status** (adverse events caused by urinary incontinence)
Hodnett, 1996 (Canada)	***X***	***X***			***X***						***X***			**Ø Professional practice** (adherence to practice guidelines)
Jansson, 2014 (Finland)		***X***					***X***				***X***			**+ Professional knowledge** (knowledge of mechanical ventilation) + Professional knowledge (adherence to practice guidelines, improved simulation exposure)
Jansson, 2016a, 2016b (Finland)		***X***					***X***							**Ø Professional knowledge** (hand hygiene, pneumonia prevention by staff)
Kalinowski, 2015 (Germany)	***X***	***X***												**Ø Professional practice** (nonpharmacological pain management techniques) **+ Patient health status** (pain management)
Kaner, 2003 (UK)		***X***		***X***										**+ Professional practice** (screening/adherence to protocol; patient management) **Ø Expenditures** (cost effectiveness)
Köpke, 2012 (Germany)	***X***	***X***									***X***		***X***	***Ø Professional practice*** *(decreased restraint use; adherence to guidelines)*
Lozano, 2004 (USA)	***X***	***X***					***X***				***X***			***+ Patient health status*** *(Asthma symptoms, frequency of steroid use)*
Mayou, 2002 (UK)	***X***	***X***						***X***						**+ Patient health status** (quality of life, anxiety/depression)
McDonald, 2005 (USA)	***X***		***X***	***X***					***X***	***X***				**Ø Professional practice** (nursing pain assessment) **+ Patient health status** (symptom management and quality of life) **+ Expenditures** (cost effectiveness)
Moon, 2015 (South Korea)	***X***	***X***												**Ø Patient health status** (incidence of delirium; in hospital mortality)
Murtaugh, 2005 (USA)	***X***			***X***					***X***	***X***				**+ Professional practice** (Adherence to nursing protocols; nursing assessment)
Naylor, 2004 (USA)	***X***	***X***								***X***				**+ Patient health status** (time to first hospitalization or death, quality of life, functional status)
Noome, 2017 (Netherlands)	***X***				***X***					***X***	***X***			**+ Professional knowledge** (knowledge of evidence-based practice in end-of-life care) **Ø Professional practice** (adherence to guidelines)
Pagaiya, 2005 (Thailand)	***X***	***X***		***X***			***X***							***+ Professional practice*** (prescribing medication to manage diabetes)
Parker, 1995 (USA)		***X***												**+ Professional knowledge** (knowledge of diabetes management) **Ø Professional practice** (diabetes management)
Premaratne, 1999 (UK)		***X***		***X***							***X***			**Ø Professional practice** (steroid prescribing) **+ Patient health status** (quality of life) **Ø Resource use** (attendance to emergency department, admission to hospital)
Rood, 2005 (Netherlands)	***X***					***X***			***X***					**+ Professional practice** (adherence to guidelines)
Ruijter, 2018 (Netherlands)	***X***							***X***						**Ø Professional practice** (adherence to guidelines)
Snelgrove-Clarke, 2015 (Canada)		***X***									***X***			**+ Professional practice** (patient safety, adherence to guidelines)
Titler, 2009; Brooks, 2008 (USA)	***X***	***X***	***X***	***X***	***X***		***X***				***X***	***X***		**+ Professional practice** (adoption of practices to address pain intensity) **+ Patient health status** (pain intensity)
Tjia, 2015 (USA)	***X***	***X***					***X***						***X***	**Ø Patient health status** (toolkit awareness) **Ø Professional practice** (adherence to guidelines, antipsychotic prescribing)
Vallerand, 2004 (USA)	***X***	***X***									***X***			**+ Professional knowledge** (perceptions of pain)
Van Gaal, 2011a; 2001b (Netherlands)	***X***	***X***				***X***	***X***	***X***						**+ Patient health status** (patient safety)
VonLengerke, 2017 (Germany)		***X***						***X***		***X***			***X***	**+ Professional practice** (hand hygiene)
Weiss 2019 (USA)	***X***	***X***										***X***		**Ø Patient health status** (in-patient readmission, return visit to hospital)
Wright, 1997 (USA)		***X***												**+ Professional practice** (universal precautions)
Zhu, 2018 (China)		***X***												**Ø Patient health status** (hypertension prevention/ management)
**Total**	**27**	**33**	**5**	**12**	**4**	**2**	***11***	***8***	**4**	**9**	***14***	**3**	**3**	

+ = positive effect on outcome; Ø = no effect on outcome

Of the three categories of EPOC implementation strategies, all studies reported interventions targeted at healthcare workers (Table 3). The most frequently used strategies to target nursing guideline implementation were educational strategies (i.e., educational meetings (*n* = 33), educational materials (*n* = 27), and educational outreach visits (*n* = 12)). Audit and feedback strategies were used in 11 studies.

A wide range of educational strategies were used which highlights the challenge of classifying these interventions under the same heading (Table 4). Many studies used training sessions that included lectures, discussions, and video presentations to provide guideline information [31–36]. Other studies employed an interactive approach to educational meetings, including case study discussions, hands-on exercises in small teams, and human patient simulation scenarios [37–39]. The educational meetings ranged in duration and frequency—from single education sessions (i.e., 30-min training sessions) to more multi-phased interventions that included multiple educational meetings over time (i.e., one, 2-h meeting per month for 6 months) [40].

**Table 4:** Characteristics of reported educational strategies

Author	Mode of delivery for educational strategy	Duration	Frequency
Ammerman 2003	Training session	2 h per session	Once
Cheater 2006	Lectures and discussions, video presentations, observed role play, individual and peer feedback + Written material provided and self-study	½ day	Twice
Daniels 2005	Interactive case study discussions; hands on exercises in small teams in the development of action plans for patient self-monitoring and self-management + Small groups also discussed effective ways to communicate specific messages to different audiences	NR	NR
Day 1991	Teaching program with didactic and interactive approaches + Practical beside demonstrations	2 h	Once
Donati 2020	Interactive training + Observational data collected and discussed	3 h + 30 min	Once + every 3 months
Elliott 1997	Educational session with lectures, small group discussions, case studies and practicums	Full day	Twice
Evans 1997	Teaching sessions + Monthly visits to clinics by a full-time nurse educator	3 h	Once
Fairrall 2005, 2010	Educational outreach sessions	1–3 h	2–6 sessions
Feldman 2004	Interactive practitioner training utilized experience facilitators, as well as role-playing and audiotaping	NR	NR
Friese 2019	E-learning modules and quiz + Email reminders reinforcing content + Tailored videos based on baseline surveys	NR	Quarterly
Haegdorens 2018, 2019	Interactive training session led by experienced practicing nurses	4 h	Once
Harrison, 2000	Training program with participation of one senior primary healthcare nurse from each intervention clinic. The workshop provided detailed information about guidelines. Participants used a problem-solving exercise to define objectives to improve quality of STD management in their clinics, which they then carried out. + Follow-up sessions were held in each clinic, addressing the topics of physical examination and history taking, counseling and attitudes, and feedback of STD surveillance results + A member of the district STD team made monthly follow-up visits to each clinic to provide regular contact, and answer questions about the syndrome packets or other aspects of the training.	Full-day NR NR	Twice 3 Monthly
Hodl 2019	Instructional meeting + Recommendations and supplementary documents (both hardcopy and PDF formats)	1 h	Once
Hodnett 1996	Workshop including lectures, panel discussions, role playing, small group discussions and audio-visual exhibits	NR	NR
Jansson 2014	Human patient simulation (HPS) education with scenario + Verbal feedback + Structured debriefing	20 min with 10-min scenario 60-min structured debriefing	Once
Jansson 2016a, 2016b	Human patient simulation (HPS) education with scenario + verbal feedback + structured debriefing	20 min with 10-min scenario 60-min structured debriefing	Once
Kalinowksi 2015	Education program (seminar with oral presentations, exercises and discussions) + Printed short summary of the clinical practice guideline	6 h	once
Kaner 2003	During outreach visit to the practice, nurses received the screening and brief alcohol intervention (SBI) program plus training on how to use the program. Two weekly telephone calls which provided support and advice about SBI.	Mean duration: 34 min	Once
Kopke 2012	Structured education program for all nursing staff + External structured intensive training workshop for nominated key nurses from different nursing homes + Printed supportive material (guideline’s 16-page short version, flyer for relatives, posters)	Intensive training workshop 1 day	Once
Lazono 2004	Workshops + Central support by an educational coordinator + An ongoing network for peer leaders via national and local teleconferences + Each leader received a tool kit containing the guidelines, key targets for behavior change, supporting reference articles, laminated pocket cards summarizing the approach to diagnosis and treatment, and academic detailing sheets on prescribing, trigger control and specialty referral + A tool kit of patient educational materials was also provided to each practice + The educational coordinator attempted to contact each leader every 1 to 2 months to provide ideas, materials and support; identify and resolve barriers to change; and encourage less active leaders.	NR	Two workshops
Mayou 2002	Trained and supervised by the researchers + Treatment was specified in a handbook	NR	NR
McDonald 2005	Information package via email with guideline details + Outreach by a Clinical Nurse Specialist who served as an “expert peer.” Standard email message from CNS one week after the first email and reminded the nurse that the CNS was available for consultation	NR	NR
Moon 2015	Training sessions and educational material	30 min	2 sessions
Murtaugh 2005	Information package via email with guideline details + Outreach by a Clinical Nurse Specialist who served as an “expert peer”. Standard email message from CNS one week after the first email asking about the status of the eligible patient, whether the HF self-care guide was useful, and whether there was a patient issue the nurse would like to discuss with the CNS.	NR	NR
Naylor 2004	Orientation and training program on guideline content	2 months	Once
Noome 2016	Educational meetings for the implementation leaders (two nurses in each ICU were chosen as the implementation leaders)	1 day	Twice over 9 months
Pagaiya 2005	Workshop with lectures, group discussions, role play and presentations + Educational outreach visit by nurse practitioners	3 days	Once
Parker 1995	Educational program of lecture format followed by a question-and-answer period	20-min sessions	7 sessions conducted 2 weeks apart
Premaratne 199	Nurse specialists provided teaching sessions on core elements of asthma care to all practice nurses + Outreach visits by the nurse specialists to help the practice nurse organize the clinic in keeping with their teaching, and assist them in improving the management of their patients.	NR	6 sessions
Rood 2005	Computer-based version of guideline – received guideline information via the clinical information system + Paper based-version of guideline, 4-page flow chart that directs nurse to relevant guideline advise	NR	NR
Rejuiter 2018	Computer based e-learning program + Tailored advice	6 months	NR
Snelgrove-Clarke 2015	Educational meetings + Personalized feedback by individualized coaching	2 h NR	Monthly Monthly
Titler 2009; Brooks 2008	Continuing Education program for senior administrative leaders+ Train the trainer program: education of nurse opinion leaders and change champions + Education of nursing and medical staff via a web-based course + Advanced practice nurse outreach every 3 weeks as consultant to nurses and physicians + Teleconferences to discuss issues, strategies for overcoming perceived barriers, progress made in education of staff, and revision of policies and documentation forms	60 min 3 days NR NR NR	Once Once NR NR Monthly
Tjia 2015	Mailed toolkit	n/a	Once
Vallerand 2004	Lecture and discussions + Packet of information + Role-playing and assertiveness training + Principal investigator (an expert consultant) was available by pager to provide support to nurses	4 h	Once
van Gaal 2011a, 2011b	Educational meeting + Case discussions on every ward + Educational materials via CD ROMs	1.5 h 30 min	Once Twice
von Lengerke 2017	Tailored educational training for nurses + feedback discussions (from clinical managers and head nurses)	NR	NR
Weiss 2019	Mandatory training	NR	NR
Wright 1997	Computer assisted intervention that presented several patient scenarios	NR	NR
Zhu 2018	Training program study to enhance the nurses’ decision-making	36 h	NR

A majority of studies (*n* = 23/41) used implementation strategies not included in the EPOC taxonomy (Table 5), which consisted of the following: adaptation of practice guidelines to local context (*n* = 9), external facilitation (*n* = 14), and changes to organizational policy (*n* = 3). In addition, a sample of the included studies also reported details of the development and delivery of specific implementation strategies, indicating participatory co-creation approach during implementation (*n* = 3 studies). Table 5 outlines these four additional implementation strategies and compares them to the EPOC taxonomy.

**Table 5:** Comparison of additional implementation strategies to EPOC taxonomy

Additional implementation strategy	Definition	Primary study implementation strategy description	Closest corresponding EPOC definition	Inclusion in other taxonomies	Comparison
**Changes to organizational policies**	Creating/adapting new policies and or adaptations or modifications to existing organizational policies to enable the implementation of evidence at a systems-level [41]	**Weiss 2019:** Added components into intervention units’ operational procedures for hospital discharge **Titler 2009:** Revision of institution-specific documents (e.g., documentation forms, policies, and procedures)	**Organizational culture:** “Strategies to change organizational culture”	ERIC Taxonomy [42]: **1. Mandate change;** Have leadership declare the priority of the innovation and their determination to have it implemented **2. Alter patient/consumer fees:** Create fee structures where patients/consumers pay less for preferred treatments (the clinical innovation) and more for less-preferred treatments **3. Change accreditation or membership requirements:** Strive to alter accreditation standards so that they require or encourage use of the clinical innovation. **Dynamic Adaption process and EPIS implementation conceptual model:** Policies, funding/resources are classified at the system-level and differ from culture/climate [41]	Organizational culture is defined as the “attitudes, experiences, beliefs, and values of the organization, acquired through social learning, that control the way individuals and groups in the organization interact with one another and with parties outside it.” [43] The “Changes to organizational policies” strategy identified in our review differs from this definition of culture, as it relates specifically to changing practice policies and procedures to implement evidence into practice. Making changes to organizational policies is not the same as interventions to change the attitudes, experiences, beliefs, and values of an organization. As such, it requires a distinct strategy, similar to the way it has been described in other implementation science research, such as the ERIC taxonomy. While organizational policies may contribute to creating organizational culture in the long term, their goal is to provide concrete direction to staff about practice/behavior, not to simply change beliefs or attitudes.
**Participatory approaches**	Collaborative research approaches, such as engaged scholarship, integrated knowledge translation, co-production, participatory action research, that engage knowledge users (e.g., patients, health care providers, policy-makers) throughout the research process [44]	**Von Lengerke 2017:** Involved medical psychologists and performed in coordination with the leading Hospital Epidemiology Department and the health economists involved in the project. **Tjia 2015:** An interdisciplinary team of geriatric physicians, pharmacists, and a nurse used the needs assessment results, CERSG data, and an environmental scan of existing NH quality improvement toolkits to develop the NH antipsychotic prescribing toolkit. **Köpke et al. 2012:** Multidisciplinary guideline development group of nationwide experts from all relevant fields, including a residents’ representative, was convened. Group members received a 1-day introduction to evidence-based medicine and guideline development. The guideline development group met 5 times between October 2007 and May 2008.	**Tailored interventions:** “Interventions to change practice that are selected based on an assessment of barriers to change, for example through interviews or surveys.”	ERIC Taxonomy [42]: **Develop academic partnerships:** Partner with a university or academic unit for the purposes of shared training and bringing research skills to an implementation project	The collaborative research approaches described in the literature involve knowledge users throughout the research process (i.e., research question generation, data collection, analysis, interpretation of findings). The approaches described in the included studies of this review highlight multidisciplinary teams involved in the research process to support guideline implementation. This differs from the “Tailored Interventions” strategy in the EPOC taxonomy, as it focuses on the *who and how* knowledge users were involved in the research process, beyond an assessment of barriers and selection of strategies.
**Facilitation**	Facilitation “represents the active ingredient of implementation, with individuals defined as facilitators taking on a change agency role to identify elements of evidence and context that might influence implementation and then utilizing appropriate facilitation methods and processes to enable the implementation process.” [45]	**Snelgrove-Clarke 2015:** Supported by the principal investigator facilitator, groups of four to six nurses participated in monthly, 2-h Action Learning meetings by sharing their experiences of adhering to the IA component of the guideline for low-risk laboring women. The facilitator conducted 1:1 coaching at least once on the birthing unit between monthly meetings. **Vallerand 2004:** The principal investigator, an expert consultant, was available by pager to provide a way for the nurses to have their questions answered while in the field. The consultant also was available to provide guidance while nurses in the clinical setting developed care plans and to direct role-playing to prepare for situations requiring advocacy for more effective pain management (e.g., telephone calls to physicians requesting changes in analgesic orders) **Feldman 2004:** The interactive practitioner training utilized experienced facilitators, as well as role-playing and audiotaping, to help nurses increase their skills in communicating with and motivating their patients to adhere to treatment instructions.	**Local opinion leaders:** “The identification and use of identifiable local opinion leaders to promote good clinical practice.”	ERIC taxonomy: **Facilitation:** A process of interactive problem solving and support that occurs in a context of a recognized need for improvement and a supportive interpersonal relationship	Facilitation has been tested in several trials as a distinct and effective implementation strategy to optimize the implementation of evidence into practice [46–49]. It differs from the “Local Opinion Leaders” category, as it focuses on distinct facilitation processes to enable change.
**Guideline adaptation**	Guideline adaptation includes reviewing the available evidence, contextualizing the evidence to the local context, and customizing recommendations to adapt guideline to the local context [50].	**Fairall 2005, 2010:** The guideline was adapted from WHO’s PAL guideline after consultation with South African primary care physicians, nurses and managers, and harmonized with local guidelines such as the national essential drug list, HIV and tuberculosis programmes. **Feldman 2004:** The HOME Plan adapted the heart failure guideline developed by the Agency for Healthcare Research and Quality (AHRQ), to the home care setting. **Harrison 2000:** These guidelines include a wall chart and a manual on the syndromic management of STD, adapted from the World Health Organization recommendations on STD treatment, and evaluated locally to determine treatment effectiveness. **Naylor 2004: E**vidence-based protocol, guided by national heart failure guidelines and designed specifically for this patient group and their caregivers with a unique focus on comprehensive management of needs and therapies associated with an acute episode of heart failure complicated by multiple comorbid conditions.	**Tailored interventions:** “Interventions to change practice that are selected based on an assessment of barriers to change, for example through interviews or surveys.” **Local consensus process:** “Formal or informal local consensus processes, for example agreeing a clinical protocol to manage a patient group, adapting a guideline for a local health system or promoting the implementation of guidelines.”	ERIC Taxonomy: **Promote adaptability:** Identify the ways a clinical innovation can be tailored to meet local needs and clarify which elements of the innovation must be maintained to preserve fidelity	Guideline adaptation differs from “Tailored Interventions”, as it focuses on tailoring and making changes to the guideline itself to meet local needs and local context, which aligns with the ERIC taxonomy “Promoting Adaptability” category. In contrast, the EPOC taxonomy category of “Tailored Interventions” focuses on tailoring the implementation strategy to the local context. Guideline adaptation also differs from “Local Consensus Process”, as it involves a more robust process to adaptation beyond what is implied in the EPOC definition of local consensus process. Guideline adaptation includes reviewing the available evidence, contextualizing the evidence to the local context, and customizing recommendations to adapt guideline to the local context [50]. Similarly, this aligns with the ERIC taxonomy “promoting adaptability” category.

### Implementation strategy effects

One hundred and two outcomes were measured across the 41 studies. The most common outcomes were professional practice (*n* = 49), followed by patient health status (*n* = 26), professional knowledge (*n* = 14), expenditure (*n* = 8), resource use (*n* = 5). The majority of outcomes (60%) were reported as positive and significant, including 64% (*n* = 9/14) of professional knowledge outcomes, 59% (*n* = 29/49) of professional practice outcomes, 54% (*n* = 14/26) patient health status outcomes, 80% (*n* = 4/5) of resource use outcomes, and 63% (*n* = 5/8) of expenditure outcomes. A summary of study outcomes is reported in Table 3.

We grouped the implementation strategies into five mutually exclusive categories to provide a narrative synthesis of study results. We created a sixth non-mutually exclusive category to describe multi-component strategies that were composed implementation strategies not included in EPOC taxonomy.

#### Educational meetings alone

Seven studies evaluated educational meetings alone [35, 39, 40, 51–54]. Five studies reported positive and significant effects on professional practice outcomes [39, 40, 51, 53, 54] and one study reported no effect [51]. Two studies reported positive and significant effects on professional knowledge [51, 52] and two studies reported no effects on patient health status outcomes [35, 39].

#### Distribution of educational materials and educational meetings plus other interventions

Twenty studies involved distribution of educational materials and educational meetings [31–34, 36, 55–69]. Of these studies, 10 examined professional practice outcomes, 11 examined patient health status outcomes, three examined professional knowledge, and one study examined resource use and expenditures (Table 3). Thirteen studies [32, 33, 36, 55–61, 63–65, 67] reported positive outcomes (*n* = 13) in all five outcome categories. All positive outcomes reported were statistically significant except two [58, 65]. Mixed effects were reported on the remaining ten outcomes.

#### Distribution of educational materials plus other interventions except educational meetings

Eight studies involved distribution of educational materials plus other interventions except educational meetings [70–78]. Of these studies, two examined professional knowledge, seven examined professional practice outcomes, one study examined patient health status outcomes and resource use, and three studies examined expenditures (Table 3). One study reported positive and significant outcomes in professional knowledge [77], four studies reported positive and significant outcomes in professional practice [70, 72, 73, 76], and varying effects were found on resource use and expenditure outcomes [70].

#### Educational meetings and other interventions except distribution of education materials

Five studies evaluated use of an educational meetings plus other interventions but did not distribute educational materials. One study reported professional knowledge outcomes, four examined professional practice outcomes, two examined patient health status outcomes, and one examined resource use. Of these studies, positive and significant effects were reported on one professional knowledge outcome [37], two professional practice outcomes [37, 79, 80], and positive but nonsignificant effects were reported on one patient health status outcome [80]. Three studies reported varying effects on professional practice [38], patient health status outcomes [81], and resource use [80].

#### Other interventions that did not include educational meetings or distribution of educational materials

Only one study evaluated other implementation strategies that did not include educational meetings or educational materials. Charrier et al. [82] evaluated two implementation strategies—audit and feedback and external facilitation and reported positive and significant effects on professional practice.

#### Interventions not included in EPOC taxonomy and educational interventions

Twenty-eight studies included a combination of educational strategies and other implementation strategies not included in EPOC taxonomy: adaptation of practice guidelines to local context (*n* = 9), external facilitation (*n* = 14), and changes to organizational policy (*n* = 3) (Table 3; Table 5). In addition, three studies also reported details of the development and delivery of specific implementation strategies, indicating participatory co-creation approach during implementation (*n* = 3) (Table 3; Table 5). Of these 23 studies, 26/43 outcomes were reported as positive (*n* = 26, 60%). More specifically, four studies reported positive and significant effects on professional knowledge, ten studies reported positive and significant effects on professional practice, seven studies reported positive and significant effects on patient health status outcomes, and five studies reported positive outcomes on resource use and expenditure outcomes (Table 3).

## Discussion

### Summary of evidence

We synthesized the findings from 41 studies (reported in 46 papers) on guideline implementation strategies for nursing practice. Multi-component educational interventions were most commonly used and included a combination of educational materials, educational meetings, and educational outreach (*n* = 36). Studies evaluating single implementation strategies focused on educational meetings alone (*n* = 5) or audit and feedback (with no educational component) (*n* = 1). Outcomes pertained to professional knowledge, professional practice, patient health status outcomes and less frequently, health system outcomes (resource use and expenditures). Given the combination and permutations of implementation strategies and outcomes, we were limited in the comparisons we could analyze. Meta-analysis was not possible owing in part to the heterogeneity among studies, including differences in implementation strategy content, mode of delivery, duration, and frequency, as well as outcomes collected (Table 3; Table 4). An important finding was that 56% of studies employed implementation strategies that are not included in the EPOC taxonomy, including the use of external facilitation (*n* = 14), multifaceted participatory approaches (*n* = 3), adaptation of practice guidelines to local context (*n* = 9), and changes to organizational policy (*n* = 3) (Table 5).

Our analysis suggests that educational meetings, in combination with other educational strategies (i.e., materials, outreach visits), are highly used in nursing and likely an effective implementation strategy for guideline implementation in nursing. Distribution of educational materials alone is effective but may not be sufficient to impact outcomes. The majority of studies (*n* = 40) evaluated educational interventions on professional knowledge outcomes (*n* = 14), professional practice outcomes (*n* = 48), patient health status outcomes (*n* = 26), and resource use/expenditure outcomes (*n* = 13). Overall, positive effects were found on the majority of professional practice outcomes (*n* = 29, 59%), professional knowledge outcomes (*n* = 9, 64%), patient health status outcomes (*n* = 14, 54%), resource use outcomes (*n* = 4, 80%), and expenditure outcomes (*n* = 5, 63%). Multi-component implementation strategies composed of interventions not included in the EPOC taxonomy (i.e., participatory approaches, facilitation, changes to organizational policies) also demonstrated positive trends on professional knowledge, professional practice, patient health status, resource use, and expenditure outcomes. Of the 43 outcomes measured with these participatory-based implementation strategies, 26 were reported as positive (*n* = 26, 60%).

Our findings on effective educational implementation strategies are in line with previous reviews of guideline implementation strategies in medicine [22], pharmacy [16], rehabilitation [13], and physiotherapy [83]. A previous review of knowledge translation interventions for promoting evidence-informed decision-making among nurses found that almost all studies identified in their review included an educational component [11]. The primary focus on educational implementation strategies assumes that nurses and other health care providers do not implement guidelines because they do not have the appropriate knowledge (i.e., barrier to guideline use is lack of knowledge of the guideline). However, many behavioral determinants, including but not limited to, an individual or group of individual’s motivation, practice context, and social influences affect the implementation process and outcomes [84]. A substantive body of implementation science research has identified modifiable behavioral determinants and/or contextual mechanisms related to implementation in health care [85, 86]. To move beyond educational implementation strategies in nursing, the field needs to be assessing barriers to guideline use, including the professional and organizational barriers to change, and use this assessment to tailor interventions to the identified barriers [87, 88].

*Quality of the evidence.*

The field of nursing is producing good quality trials. In this review, over two-thirds of studies (*n* = 29, 70%) were of high quality (as indicated by protection against contamination) with a low risk of bias. Comparatively, a previous review of guideline implementation studies in medicine [22] found 54.5% to be of high quality and 4.5% low quality (RCT *n* = 110). Despite the differences in the number of studies in medicine compared with nursing, the quality of the studies appears to be similar in both fields. Other reviews on similar topics among allied health professions and nursing report low methodological quality of the analyzed studies; however, these reviews included non-randomized control trials, quasi-experimental, and/or observational studies [11, 13, 16].

Reporting continues to be an issue identified in this review, as was also identified in previous systematic reviews in medicine [22], allied health [13, 15, 16, 83], and nursing [19]. Study quality in the nursing implementation field revealed that 25% of the risk of bias indicators were rated as unclear. Many factors are considered when assessing the methodological quality of included RCT studies such as risk of contamination, concealment of allocation, blinded assessments of outcomes, baseline measures, and follow up of professionals. Unfortunately, the documentation on how these issues are managed in a particular study is often less than adequate, thereby making it difficult to ascertain if it is inadequate reporting or inadequate trial procedures. Reporting guidelines exist for trials and intervention description (EQUATOR Network [89]), including CONSORT statements [90] and the TIDiER guidelines [91]. While some studies in this review preceded release of reporting guidelines, only few provided adequate intervention description that aligns with reporting guidelines for interventions. It was difficult to discern intervention dose to support replicability. Future implementation intervention studies should use reporting guidelines to clearly articulate intervention components and strengthen the evidence base on guideline implementation in nursing.

### Implementation strategies in nursing

This review highlights several key discoveries for others involved in implementation work in nursing. First, nursing appears to be advancing the evidence base on implementation strategies. There is an increasing number of RCTs in recent years and over half (*n* = 23) of the studies included guideline implementation strategies not described in the EPOC taxonomy, including facilitation (*n* = 14), guideline adaptation to the local context (*n* = 9), changes to organizational policies (*n* = 3), and participatory approaches to research (*n* = 3) (Table 5). A growing body of evidence, stemming from the nursing literature, reports facilitation as an effective strategy to optimize the implementation of evidence into practice [46–48]. A systematic review of guideline implementation in primary care found that practices supported by facilitators were 2.76 times more likely to adopt evidence-based clinical practice guidelines [46]. Further, guideline adaptation to local context is an implementation strategy that relates to the planned action phases [88, 92, 93] and highlight efforts to align an implementation strategy to the local context and build on existing knowledge locally about effective strategies to increase uptake of evidence-based practice [92]. Similarly, previous research has shown participatory research approaches, that focus on producing knowledge and implementing evidence that is relevant to the needs of knowledge users, is an important strategy to consider when implementing evidence into practice [44].

Many of the identified implementation strategies align with the existing implementation science frameworks. Leeman et al. [94] offer a five-component classification system for implementation strategies, including dissemination strategies, implementation process strategies, integration strategies, capacity-building strategies, and scale-up strategies. The more prevalent educational implementation strategies identified in this review align with the implementation process strategies category. Our additional strategies that are not included in the EPOC taxonomy (facilitation, participatory approach, adaptation of guidelines, and changes to organizational policies) align with the integration, capacity-building, and scale-up strategies. Further, the additional implementation strategies map onto the Consolidated Framework for Implementation Research (CFIR) framework [95] which illustrates how these strategies target known behavioral and contextual determinants, including the intervention characteristics, process, and inner setting domains. This differs from educational implementation strategies that target the characteristics of the individual (i.e., knowledge and beliefs about the intervention). By mapping the identified implementation strategies onto existing frameworks, it is clear that multi-component educational and participatory strategies are useful to address multiple stages of the implementation process, and target multi-level behavioral and contextual determinants of guideline implementation.

Identification of these additional implementation strategies is an important finding for nursing implementation research and practice and may help to move beyond traditional educational approaches to implementation. Facilitation, guideline adaptation, changes to organizational policy, and participatory approaches are strategies that target guideline implementation within teams, units, or organizations. In a nursing context of 24/7 care, the decision-making process often occurs in a team or group context, as well as with individual practitioner decision-making [18, 96]. This differs from previous reviews of implementation strategies in medicine, pharmacy, and dentistry where individual-based implementation strategies are singularly used (i.e., reminders, audit and feedback). These may not function in the same way within a team context. The concern is that by only categorizing implementation strategies similar to reviews with other health care providers, we may be missing an opportunity to understand how implementation works in nursing contexts. To this point, the majority of included nursing trials (*n* = 23/41) used implementation strategies not included in the EPOC review and showed positive impacts on patient, provider, and health system outcomes.

Overall, implementation researchers and practitioners may find results of our systematic review helpful moving forward. First, for those undertaking similar reviews in the future, there are other taxonomies that capture more of the strategies we identified through our inductive thematic analysis. Numerous taxonomies and classification schemes have been published that describe a range of implementation strategies [97]. For example, the Expert Recommendations for Implementing Change (ERIC) project provides a compilation of implementation strategies, including strategies such as facilitation, promote adaptability, assess for readiness, identify barriers and facilitators, and develop academic partnerships [42]. Further, Slaughter et al. 2017 [98] provide practical resources for implementation researchers that includes a variety of classification schemes for knowledge translation interventions. Nursing implementation researchers should aim to explore the effectiveness and feasibility of these additional types of interventions in future work. Second, nurses in practice settings or other disciplines that work primarily in teams may benefit from using a taxonomy that includes team-based implementation strategies to plan and execute their implementation projects.

A second important discovery is that pre-existing search strategies for implementation strategy literature in other health professions does not work for locating guideline implementation studies in the nursing literature. We duplicated the search strategies described in a previous review of implementation strategies used in medicine [28]; however, these strategies did not locate any relevant nursing literature. We then crafted our own extensive search strategy and located the papers included for this review. This may be related to the medicine-focused search strategy not including CINAHL or other nursing databases (e.g., Proquest Nursing and Allied Health or British Nursing Index) and not including all types of common nursing roles (e.g., registered nurses). As such, efforts are needed to go beyond pre-existing search strategies and taxonomies to capture nursing trials and the strategies employed. The nursing implementation science field could be advanced by a separate review of non-RCT studies to understand a broader base of implementation strategies. Findings from this present review highlight that the state of science is not mature enough for solely analyzing RCTs; useful information from other types of studies would supplement findings from RCTs. The current review provides the start of a search framework to be used and expanded on by others to explore additional guideline implementation strategies in nursing in quasi or non-experimental research designs and qualitative research studies.

### Future research directions

A key finding from this review is the number of studies (*n* = 23) that used participatory-based implementation strategies (Table 3; Table 5). Unfortunately, due to heterogeneity of implementation strategies, direct comparisons between the 13 studies that only included EPOC taxonomy implementation strategies versus these 23 studies is not possible. Future research is needed to explore the effectiveness of these types of implementation strategies not included in the EPOC taxonomy. This will help to move this field beyond educational interventions and understand how different components of these strategies work in a nursing context and with what effect. Further, it will help practitioners select the most appropriate, feasible, and effective implementation strategies for their specific nursing context. As previously discussed, we recommend examining these implementation strategies in the context of descriptive and qualitative studies to understand what works, for whom, and in what context.

Importantly, the implementation science literature generally recommends the use of theory to guide intervention design and implementation. Use of theory supports development of interventions that target behavioral determinants and lead to potentially stronger effects [84, 99]. Further, theory use leads to evaluations that are more robust in developing a theoretical understanding of intervention effects [84]. Despite its benefits, to date, reviews in medicine and other allied health professions have not reported extensive use of theory in intervention design. In a review of guideline dissemination and implementation strategies in the cancer care context, only one of 33 included studies used theory to directly inform the design of the intervention [100]. Similarly, only 14 out of 158 studies included in a review of uptake of evidence-based interventions in maternity care reported the use of theory [101]. Notably, in the current review, 40% of studies used theory to inform the design of interventions including studies. It appears that nurses involved in implementation research are early adopters, with the use of theory in intervention design dating back to included studies published in 1997 [56]. This may be explained by the strong theoretical underpinning in nursing and the use of theory to inform nursing practice [102]. Regardless, this warrants further investigation to understand how theory is being used and what is its effect on implementation strategy development and outcomes in nursing.

### Limitations

The following limitations of this systematic review should be considered. First, we used a broad definition of guidelines so our interpretation may differ from others. Second, only studies published in English were included and potentially relevant studies published in other languages may have been missed. Third, many papers lacked detail on the implementation strategies used, which made it challenging to synthesize similar strategies and understand the duration and frequency needed to have the desired effect. Lastly, we had hoped to be able to do a meta-analysis; however, a narrative review was conducted because the methodological and clinical heterogeneity of the studies in this review revealed that meta-analysis was not appropriate. This level of heterogeneity among implementation studies has also been found in similar reviews [16, 100] of implementation strategies and speaks to the need for further work in the field to understand implementation effectiveness and improve reporting.

## Conclusions

In this review, we aimed to describe the use and effects of implementation strategies to facilitate the uptake of guidelines focused on nursing care. While the evidence is limited, multi-component educational strategies were most commonly tested. Implementation strategies that include educational strategies reported promising effects on professional knowledge, professional practice, patient health status, and resource use/expenditure outcomes. We discovered additional implementation strategies in these studies that are not currently included in the EPOC taxonomy. Strategies such as facilitation, guideline adaptation, using a participatory approach, and changing organizational policies may be useful for both nurses and others similarly working in teams in health care to implement practice guidelines. Future implementation research in nursing, using qualitative, quantitative, and mixed methods research designs, is needed to understand what works, for whom and in what context. This includes assessing barriers to guideline use and tailoring implementation strategies to identified barriers. Ultimately, these research efforts will help to strengthen the evidence on effective guideline implementation strategies in nursing practice. While not the purpose of our review, we have determined that implementation of guidelines by nurses, on balance, tends to improve the process of care, patient health outcomes and health system outcomes (resource use and costs). Hence, the need to continue studying how to *effectively* encourage adoption of guidelines in nursing is vital.

## Supplementary Information

**Additional file 1:** Deviations from a priori protocol

**Additional file 2:** Search strategy

**Additional file 3:** PRISMA Checklist

## Data Availability

All data generated or analyzed during this study are included in this published article [and its supplementary information files].

## Abbreviations

RCT: Randomized controlled trials
cRCT: Cluster randomized controlled trials
APN: Advanced practice nurse
NP: Nurse practitioner
CE: Clinical educators
CNS: Clinical nurse specialist
LPN: Licensed practical nurse
RPN: Registered practical nurse
RN: Registered nurse
EPOC: Effective practice and organization of care
