# Supplementary material for: Use and effects of implementation strategies for practice guidelines in nursing: a systematic review
Source: Implement Sci. 2021 Dec 4;16:102. doi: 10.1186/s13012-021-01165-5 (PMC8642950; doi:10.1186/s13012-021-01165-5)
Supplement: Supplementary file 1 — Additional file 1: Deviations from a priori protocol [file 13012_2021_1165_MOESM1_ESM.docx]

**Supplemental File 1 – Deviations from a priori protocol**

| **Protocol Item** | **A priori Methods** | **Final Review Methods** | **Rationale for Deviation from Protocol** |
| --- | --- | --- | --- |
| **Research Questions** | - To determine the effectiveness of different practice guideline implementation interventions on nursing professional practice and/or patient health outcomes.   - Comparison 1: Guideline implementation intervention versus usual care.   - Comparison 2: Guideline implementation intervention A versus guideline implementation intervention B | - To determine the effectiveness and feasibility of implementation strategies to facilitate the uptake of guidelines focused on nursing care | - Methodological and clinical heterogeneity of these studies determined that a meta-analysis was not suitable. |
| **Eligibility Criteria** | - ***Study Design***: Randomized controlled trials (RCTs) - ***Language:*** English - ***Participants***: All different categories of nurses - ***Interventions***: intervention used to implement a guideline into nursing or a multidisciplinary team with a focus on nursing outcomes - ***Comparators:*** “usual practice” or “usual care” which indicates no implementation intervention was used to change nursing practice - ***Outcomes:*** process or outcome of care provided by nursing professionals (i.e, professional knowledge, professional practice, patient health outcomes or resource use/expenditure outcomes) | ***Same as a priori methods*** |  |
| **Search Strategy** | - Built on an existing EPOC search strategy, using search terms and medical subject headings (MeSH) relevant to “clinical guidelines” AND “implementation” AND “nursing” AND “randomized controlled trial” - Five databases (Medline, EMBASE, CINAHL, PsycINFO, AMED) and the Cochrane EPOC registry - No restrictions were placed on the search - Also used the search strategies developed by Grimshaw and colleagues for their investigation of the effectiveness and efficiency of guideline dissemination and implementation strategies in the context of medicine, adjusted to focus on nursing and rerun against MEDLINE, CINAHL and EMBASE. Scanned reference lists of papers identified for inclusion for any additional references not captured. | ***Same as a priori methods*** |  |
| **Study Selection** | - Two reviewers will independently screen the titles and abstracts and full-text articles against inclusion criteria | ***Same as a priori methods*** |  |
| **Data Abstraction** | - Two reviewers will independently abstract data from included studies using a standardized form adapted from the EPOC data collection checklist: a) study design, b) participants, c) setting, d) data collection methods, e) practice guideline, f) types of implementation strategies, g) outcome measures, and h) study results. | ***Same as a priori methods*** |  |
| **Categorization of Implementation Strategies** | - Implementation strategies will be classified using the EPOC taxonomy for implementation interventions | - First, we deductively categorized strategies into the EPOC taxonomy’s section on implementation strategies with three sub-categories: (i) Interventions targeted at healthcare organizations, (ii) Interventions targeted at healthcare workers, and (iii) Interventions targeted at specific types of practice, conditions or settings. - Second, for those strategies not included in the EPOC taxonomy, we used an inductive thematic analysis approach to inductively categorize these strategies and generate additional implementation strategy categories | - We identified additional implementation strategies being used in nursing trials that were not included in the EPOC taxonomy |
| **Study Quality** | - Two reviewers independently assessed the risk of bias using the EPOC Risk of Bias 2.0 checklist in Covidence | ***Same as a priori methods*** |  |
| **Data Analysis** | - Dichotomous and continuous outcome variables will be analysed separately. - Crude risk differences (CRD) will be calculated by comparing post intervention values for all of the outcomes. - Adjusted risk difference (ARD) values will be calculated as change between baseline and post-intervention values when the baseline values of both the control and intervention group are provided. - We will report effect sizes of the continuous outcomes (both CRD and ARD) as relative effects rather than absolute effects since we will adjust the change relative to baseline of the control group. - Effect sizes of the dichotomous outcomes will not be adjusted for baseline and thus, reported as absolute effects. - Each of the outcome categories of interest will be analyzed separately by calculating median effect size. - A positive risk difference (RD) indicates that both the professional and patient health outcome demonstrated a greater improvement, used less resources, or incurred a lower cost in the intervention group than the control group. - All studies will be included regardless of level of risk of bias. - For the dichotomous outcome variables, CRD will be calculated as the percentage post intervention differences between intervention and control group: (**Post**_intervention_ – **Post** _control_) - We will calculate the ARD as the percentage difference in post intervention differences in mean and the baseline difference in mean: (**Post**_intervention_ – **Post** _control_) – (**Baseline**_intervention_ – **Baseline** _control_) - For continuous outcomes, we will calculate the CRD as the difference between the mean of intervention and control group after the intervention divided by the post control group mean: (**Post**_intervention_ – **Post** _control_) ÷ **Post**_control_ - We will calculate the ARD as the post intervention difference in means minus the baseline difference in means and divide that by the baseline control group mean: [(**Post**_intervention_ – **Post** _control_) – (**Baseline**_intervention_ – **Baseline** _control_)] ÷ **Baseline**_control_ | - A narrative synthesis to describe implementation strategies and their effectiveness in achieving outcomes for guideline implementation in nursing - Descriptive statistics were used to generate mean number of implementation strategies used in each category. - The frequency of each EPOC taxonomy category was reported. - Separate comparisons were made for categories of implementation strategies and compared to grouped study results to determine whether they were related to positive and significant improvement in professional knowledge outcomes, professional practice outcomes, patient health outcomes, resource use outcomes, and expenditure outcomes. | - The a priori methods generated a heterogeneous set of papers with a wide variety of implementation strategies and outcome measures. - It was challenging to make sense of the data in a useful way as planned to move the state of the science forward. - As such, we made a decision to conduct a narrative review of the included papers with a different lens to describe the findings in a more useful way. |
