# Supplementary material for: Use and effects of implementation strategies for practice guidelines in nursing: a systematic review
Source: Implement Sci. 2021 Dec 4;16:102. doi: 10.1186/s13012-021-01165-5 (PMC8642950; doi:10.1186/s13012-021-01165-5)
Supplement: Supplementary file 2 — Additional file 2: Search strategy [file 13012_2021_1165_MOESM2_ESM.docx]

**Supplemental File 2 – Search Strategy**

**MEDLINE**

1. Critical Pathways/

2. exp Clinical Protocols/

3. exp "guideline [publication type]"/

4. Evidence-Based Medicine/

5. Guideline adherence/

6. practice guideline$.mp.

7. (research$ adj3 practice$).mp. [mp=title, original title, abstract, name of substance word, subject heading word]

8. (research$ adj3 (guideline$ or protocol$)).mp. [mp=title, original title, abstract, name of substance word, subject heading word]

9. (research based adj3 (care or practice$ or method$ or protocol$)).mp. [mp=title, original title, abstract, name of substance word, subject heading word]

10. (evidence based adj3 (care or practice$ or method$ or protocol$)).mp. [mp=title, original title, abstract, name of substance word, subject heading word]

11. or/1-10

12. education/ or curriculum/ or competency-based education/ or "mainstreaming (education)"/ or problem-based learning/ or exp education, distance/ or education, nonprofessional/ or exp education, professional/ or exp inservice training/ or mentors/ or preceptorship/ or exp teaching/ or "Health Knowledge, Attitudes, Practice"/

13. exp Teaching Materials/

14. Information Dissemination/

15. exp "diffusion of innovation"/

16. Training Support/

17. mt.fs.

18. ed.fs.

19. or/12-18

20. ((educat$ or train$ or usage or using or implement$ or disseminat$ or diffusion or uptake or adopt$ or promot$ or strateg$ or intervention$) adj3 (strateg$ or method$ or means or process$ or procedur$ or technique$ or intervention$)).mp. [mp=title, original title, abstract, name of substance word, subject heading word]

21. (disseminat$ adj2 (research$ or evidence or best practice$)).mp. [mp=title, original title, abstract, name of substance word, subject heading word]

22. disseminat$.mp. [mp=title, original title, abstract, name of substance word, subject heading word]

23. (introduc$ adj2 (protocol$ or guideline$)).mp. [mp=title, original title, abstract, name of substance word, subject heading word]

24. (transfer$ adj2 (research$ or evidence$)).mp. [mp=title, original title, abstract, name of substance word, subject heading word]

25. or/20-24

26. 19 or 25

27. exp clinical trials/

28. double-blind method/ or meta-analysis/ or random allocation/

29. randomized controlled trial.pt.

30. controlled clinical trial.pt.

31. random$.tw.

32. (time adj series).tw.

33. controlled before.tw.

34. or/27-33

35. (pre-test or pretest or posttest or post-test).tw.

36. control$.tw.

37. 35 and 36

38. 34 or 37

39. animal/

40. human/

41. 39 not (39 and 40)

42. 38 not 41

43. exp health personnel/ or allied health personnel/ or exp dental staff/ or exp faculty, medical/ or faculty, nursing/ or health educators/ or exp health facility administrators/ or laboratory personnel/ or exp nurses/ or exp nursing staff/ or personnel, hospital/ or dental staff, hospital/ or nursing staff, hospital/ or pharmacists/ or exp Patient Care Team/

44. nu.fs.

45. (nurs$ or therapist$ or technician$ or team$ or dietician$ or nutritionist$ or pharmacist$ or physiotherapist$ or assistant$ or hygienist$ or pathologist$).mp. [mp=title, original title, abstract, name of substance word, subject heading word]

46. 43 or 44 or 45

47. 11 and 26 and 42 and 46

**CINAHL**

1. exp Clinical Trials/

2. Evaluation Research/ or Comparative Studies/ or Descriptive Statistics/ or exp Random Sample/ or exp Pretest-Posttest Design/ or exp QUASI-EXPERIMENTAL STUDIES/ or meta analysis/ or Minimum Data Set/

3. (random$ adj1 (allocat$ or assign$)).mp. [mp=title, subject heading word, abstract, instrumentation]

4. (research adj3 (clinical or randomised or randomized)).mp. [mp=title, subject heading word, abstract, instrumentation]

5. (study adj2 (clinical or randomised or randomized)).mp. [mp=title, subject heading word, abstract, instrumentation]

6. ((control adj2 group) or (experimental adj2 group)).mp. [mp=title, subject heading word, abstract, instrumentation]

7. clinical trial.pt.

8. controlled clinical trial.pt.

9. controlled before.tw.

10. or/1-9

11. (pre-test or pretest or posttest or post-test or pre-post test).tw.

12. (control$ or design$ or experiment$).tw.

13. 11 and 12

14. 10 or 13

15. animal/

16. human/

17. 15 not (15 and 16)

18. 14 not 17

19. Practice Guidelines/ed, ut [Education, Utilization]

20. practice guideline$.mp. or Practice Guidelines/ or practice guideline.pt.

21. professional practice, evidence-based/ or nursing practice, evidence-based/ or occupational therapy practice, evidence-based/ or physical therapy practice, evidence-based/ or professional practice, research-based/ or nursing practice, research-based/ or occupational therapy practice, research-based/ or physical therapy practice, research-based/ or intervention trials/

22. Professional Practice, Evidence-Based/ed, ut [Education, Utilization]

23. Nursing Practice, Evidence-Based/ed, ut [Education, Utilization]

24. Occupational Therapy Practice, Evidence-Based/ed, ut [Education, Utilization]

25. Physical Therapy Practice, Evidence-Based/ed [Education]

26. Professional Practice, Research-Based/ed, ut [Education, Utilization]

27. Nursing Practice, Research-Based/ed, ut [Education, Utilization]

28. Physical Therapy Practice, Research-Based/ed [Education]

29. occupational therapy practice, research-based/

30. RESEARCH/ut [Utilization]

31. Professional Knowledge/

32. Research, Speech-Language-Hearing Therapy/

33. Nursing Practice/st, ev [Standards, Evaluation]

34. Nursing Practice, Evidence-Based/

35. Clinical Competence/

36. KNOWLEDGE/ev, ut [Evaluation, Utilization]

37. exp Nursing Protocols/

38. Nursing Protocols/

39. Nursing Knowledge/

40. Protocols/ or protocol$.mp. [mp=title, subject heading word, abstract, instrumentation]

41. (research$ adj3 practice$).mp. [mp=title, subject heading word, abstract, instrumentation]

42. (guideline$ adj2 (clinical or research or "evidence based" or "research based")).mp. [mp=title, subject heading word, abstract, instrumentation]

43. (guideline$ adj2 (clinical or research or "evidence based" or "research based" or "best practice based")).mp. [mp=title, subject heading word, abstract, instrumentation]

44. (research based adj3 (care or practice$ or method$ or protocol$ or guideline$)).mp. [mp=title, subject heading word, abstract, instrumentation]

45. (research$ adj3 guideline$).mp. [mp=title, subject heading word, abstract, instrumentation]

46. (evidence based adj3 (care or practice$ or method$ or protocol$ or guideline$)).mp. [mp=title, subject heading word, abstract, instrumentation]

47. or/19-46

48. "diffusion of innovation"/ or exp nonverbal communication/

49. Staff Development/

50. education, continuing/ or education, nursing, continuing/

51. (seminar$ and workshop$).mp. [mp=title, subject heading word, abstract, instrumentation]

52. teaching/ or exp teaching materials/ or exp teaching methods/

53. exp Education, Allied Health/

54. Program Implementation/

55. Nursing Staff, Hospital/ed [Education]

56. Staff Development Instructors/

57. Personnel, Health Facility/ed [Education]

58. Marketing/

59. or/48-58

60. ((educat$ or train$ or usage or using or implement$ or disseminat$ or diffusion or uptake or adopt$ or promot$ or strateg$ or intervention$) adj3 (strateg$ or method$ or means or process$ or procedur$ or technique$ or intervention$ or "diffusion of innovation")).mp. [mp=title, subject heading word, abstract, instrumentation]

61. (disseminat$ adj2 (research$ or evidence or best practice$)).mp. [mp=title, subject heading word, abstract, instrumentation]

62. (disseminat$ adj2 (research$ or evidence or best practice$)).mp. [mp=title, subject heading word, abstract, instrumentation]

63. (transfer$ adj2 (research$ or evidence$)).mp. [mp=title, subject heading word, abstract, instrumentation]

64. disseminat$.mp. [mp=title, subject heading word, abstract, instrumentation]

65. (introduc$ adj3 protocol$).mp. [mp=title, subject heading word, abstract, instrumentation]

66. or/59-65

67. exp allied health personnel/ or exp Multidisciplinary Care Team/ or community health workers/ or health personnel, unlicensed/ or mental health personnel/ or midwives/ or nursing home personnel/ or nursing manpower/ or exp nurses/ or pharmacists/ or rural health personnel/

68. exp allied health personnel/ or exp Multidisciplinary Care Team/ or community health workers/ or health personnel, unlicensed/ or mental health personnel/ or midwives/ or nursing home personnel/ or nursing manpower/ or exp nurses/ or pharmacists/ or rural health personnel/ or exp hospital units/

69. (nurs$ or therapist$ or technician$ or team$ or dietician$ or nutritionist$ or pharmacist$ or physiotherapist$ or assistant$ or hygienist$ or pathologist$ or midwi$ or chiropod$ or speech pathologist$ or speech therap$ or physical therapist$ or dietetics or psychologist$ or radiograp$ or occupational therap$ or pharmac$ or allied health care personnel or allied health care professional$ or audiologist$ or technician$ or technologist$ or physician assistant$ or social worker$ or speech language pathologist$ or patient care team$ or multidisciplinary care team$ or para medical or paramedical or health educator$ or laboratory personnel or hospital dental staff clinical multidisciplinary team$).mp. [mp=title, subject heading word, abstract, instrumentation]

70. 67 or 68 or 69

71. 18 and 47 and 66 and 70

**EMBASE**

1. Randomized Controlled Trial/

2. "cost effectiveness analysis"/

3. Clinical Trial/

4. Major Clinical Study/

5. outcomes research/

6. Controlled Study/

7. random$.tw.

8. (time adj series).tw.

9. controlled before.tw.

10. or/1-9

11. (pre test or pretest or post-test or pre-post test).tw.

12. (control$ or design$ or experiment$).tw.

13. 11 and 12

14. 10 or 13

15. animal/

16. human/

17. 15 not (15 and 16)

18. 14 not 17

19. practice guideline/ or clinical pathway/ or clinical protocol/ or consensus development/ or good clinical practice/

20. evidence based medicine/ or meta analysis/ or outcomes research/

21. practice guideline$.mp.

22. (research$ adj3 practice$).mp. [mp=title, abstract, subject headings, heading word, drug trade name, original title, device manufacturer, drug manufacturer name]

23. (research$ adj3 (guideline$ or protocol$)).mp. [mp=title, abstract, subject headings, heading word, drug trade name, original title, device manufacturer, drug manufacturer name]

24. (research based adj3 (care or practice$ or method$ or protocol$)).mp. [mp=title, abstract, subject headings, heading word, drug trade name, original title, device manufacturer, drug manufacturer name]

25. (evidence based adj3 (care or practice$ or method$ or protocol$)).mp. [mp=title, abstract, subject headings, heading word, drug trade name, original title, device manufacturer, drug manufacturer name]

26. or/19-25

27. staff training/

28. clinical education/ or exp paramedical education/ or exp nursing education/

29. education/ or continuing education/ or curriculum/ or educational technology/ or education program/ or teaching/ or information dissemination/

30. ((educat$ or train$ or usage or using or implement$ or disseminat$ or diffusion or uptake or adopt$ or promot$ or strateg$ or intervention$) adj3 (strateg$ or method$ or means or process$ or procedur$ or technique$ or intervention$)).mp. [mp=title, abstract, subject headings, heading word, drug trade name, original title, device manufacturer, drug manufacturer name]

31. (disseminat$ adj2 (research$ or evidence or best practice$)).mp. [mp=title, abstract, subject headings, heading word, drug trade name, original title, device manufacturer, drug manufacturer name]

32. (introduc$ adj2 (protocol$ or guideline$)).mp. [mp=title, abstract, subject headings, heading word, drug trade name, original title, device manufacturer, drug manufacturer name]

33. (transfer$ adj2 (research$ or evidence$)).mp. [mp=title, abstract, subject headings, heading word, drug trade name, original title, device manufacturer, drug manufacturer name]

34. or/27-33

35. exp paramedical personnel/ or hospital personnel/ or exp nurse/

36. ((health adj2 administrator$) or (hospital adj2 administrator$)).mp. [mp=title, abstract, subject headings, heading word, drug trade name, original title, device manufacturer, drug manufacturer name]

37. (nurs$ or therapist$ or technician$ or team$ or dietician$ or nutritionist$ or pharmacist$ or physiotherapist$ or assistant$ or hygienist$ or pathologist$ or midwi$ or chiropod$ or speech pathologist$ or speech therap$ or physical therapist$ or dietetics or psychologist$ or radiograp$ or occupational therap$ or pharmac$ or allied health care personnel or allied health care professional$ or audiologist$ or technician$ or technologist$ or physician assistant$ or social worker$ or speech language pathologist$ or patient care team$ or multidisciplinary care team$ or para medical or paramedical or health educator$ or laboratory personnel or hospital dental staff clinical multidisciplinary team$).mp. [mp=title, abstract, subject headings, heading word, drug trade name, original title, device manufacturer, drug manufacturer name]

38. or/35-37

39. 18 and 26 and 34 and 38

**AMED**

1. practice guidelines/

2. exp Clinical Protocols/

3. guidelines/

4. Evidence-Based Medicine/

5. (practice guideline$ or "state of the art" or guideline$).mp. [mp=abstract, heading words, title]

6. (research$ adj3 practice$).mp. [mp=abstract, heading words, title]

7. (research$ adj3 (guideline$ or protocol$)).mp. [mp=abstract, heading words, title]

8. (research based adj3 (care or practice$ or method$ or protocol$ or guideline$)).mp. [mp=abstract, heading words, title]

9. (evidence based adj3 (care or practice$ or method$ or protocol$ or guideline$)).mp. [mp=abstract, heading words, title]

10. or/1-9

11. education/ or curriculum/ or competency-based education/ or "mainstreaming (education)"/ or problem-based learning/ or exp education, distance/ or education, nonprofessional/ or exp education, professional/ or exp inservice training/ or mentors/

12. ((educat$ or train$ or usage or using or implement$ or disseminat$ or diffusion or uptake or adopt$ or promot$ or strateg$ or intervention$) adj3 (strateg$ or method$ or means or process$ or procedur$ or technique$ or intervention$ or "diffusion of innovation")).mp. [mp=abstract, heading words, title]

13. (disseminat$ adj2 (research$ or evidence or best practice$)).mp. [mp=abstract, heading words, title]

14. disseminat$.mp. [mp=abstract, heading words, title]

15. (introduc$ adj2 (protocol$ or guideline$)).mp. [mp=abstract, heading words, title]

16. (transfer$ adj2 (research$ or evidence$)).mp. [mp=abstract, heading words, title]

17. or/11-16

18. exp clinical trials/

19. double-blind method/ or meta-analysis/ or random allocation/

20. randomized controlled trial.pt.

21. controlled clinical trial.pt.

22. random$.tw.

23. (time adj series).tw.

24. controlled before.tw.

25. or/18-24

26. (pre-test or pretest or posttest or post-test or pre-post test).tw.

27. (control$ or design$ or experiment$).tw.

28. 26 and 27

29. 25 or 28

30. exp health personnel/ or allied health personnel/ or exp nurses/ or exp nursing staff/ or personnel, hospital/ or pharmacists/ or exp Patient Care Team/ or exp hospital units/

31. ((health adj2 administrator$) or (hospital adj2 administrator$)).mp. [mp=abstract, heading words, title]

32. (nurs$ or therapist$ or technician$ or team$ or dietician$ or nutritionist$ or pharmacist$ or physiotherapist$ or assistant$ or hygienist$ or pathologist$ or midwi$ or chiropod$ or speech pathologist$ or speech therap$ or physical therapist$ or dietetics or psychologist$ or radiograp$ or occupational therap$ or pharmac$ or allied health care personnel or allied health care professional$ or audiologist$ or technician$ or technologist$ or physician assistant$ or social worker$ or speech language pathologist$ or patient care team$ or multidisciplinary care team$ or para medical or paramedical or health educator$ or laboratory personnel or hospital dental staff clinical multidisciplinary team$).mp. [mp=abstract, heading words, title]

33. or/30-32

34. 10 and 17 and 29 and 33

**psycINFO**

1. Evidence-Based Medicine/

2. treatment guidelines/

3. professional standards/

4. intervention/

5. (practice guideline$ or "state of the art" or protocol$).mp. [mp=title, abstract, heading word, table of contents, key concepts]

6. (research$ adj3 practice$).mp. [mp=title, abstract, heading word, table of contents, key concepts]

7. (research$ adj3 (guideline$ or protocol$)).mp. [mp=title, abstract, heading word, table of contents, key concepts]

8. (research based adj3 (care or practice$ or method$ or protocol$ or guideline$)).mp. [mp=title, abstract, heading word, table of contents, key concepts]

9. (evidence based adj3 (care or practice$ or method$ or protocol$ or guideline$)).mp. [mp=title, abstract, heading word, table of contents, key concepts]

10. or/1-9

11. education/ or exp inservice training/ or mentors/ or professional development/ or exp teaching methods/ or health knowledge/ or exp personnel training/ or clinical methods training/ or exp continuing education/ or nursing education/

12. Information Dissemination/

13. ((educat$ or train$ or usage or using or implement$ or disseminat$ or diffusion or uptake or adopt$ or promot$ or strateg$ or intervention$) adj3 (strateg$ or method$ or means or process$ or procedur$ or technique$ or intervention$ or "diffusion of innovation")).mp. [mp=title, abstract, heading word, table of contents, key concepts]

14. (disseminat$ adj2 (research$ or evidence or best practice$)).mp. [mp=title, abstract, heading word, table of contents, key concepts]

15. disseminat$.mp. [mp=title, abstract, heading word, table of contents, key concepts]

16. (introduc$ adj2 (protocol$ or guideline$)).mp. [mp=title, abstract, heading word, table of contents, key concepts]

17. (transfer$ adj2 (research$ or evidence$)).mp. [mp=title, abstract, heading word, table of contents, key concepts]

18. or/11-17

19. clinical trials/

20. meta-analysis/

21. random$.tw.

22. (time adj series).tw.

23. controlled before.tw.

24. (pre-test or pretest or posttest or post-test or pre-post test).tw.

25. (control$ or design$ or experiment$).tw.

26. or/19-25

27. exp health personnel/ or exp allied health personnel/ or exp nurses/ or exp management personnel/ or medical personnel/ or exp pharmacists/ or exp physical therapists/ or exp psychiatric hospital staff/ or exp teams/ or therapists/

28. ((health adj2 administrator$) or (hospital adj2 administrator$)).mp. [mp=title, abstract, heading word, table of contents, key concepts]

29. (nurs$ or therapist$ or technician$ or team$ or dietician$ or nutritionist$ or pharmacist$ or physiotherapist$ or assistant$ or hygienist$ or pathologist$ or midwi$ or chiropod$ or speech pathologist$ or speech therap$ or physical therapist$ or dietetics or psychologist$ or radiograp$ or occupational therap$ or pharmac$ or allied health care personnel or allied health care professional$ or audiologist$ or technician$ or technologist$ or physician assistant$ or social worker$ or speech language pathologist$ or patient care team$ or multidisciplinary care team$ or para medical or paramedical or health educator$ or laboratory personnel or hospital dental staff clinical multidisciplinary team$).mp. [mp=title, abstract, heading word, table of contents, key concepts]

30. or/27-29

31. 10 and 18 and 26 and 30
